# Sphingomyelin synthase-related protein generates diacylglycerol *via* the hydrolysis of glycerophospholipids in the absence of ceramide

**DOI:** 10.1016/j.jbc.2021.100454

**Published:** 2021-02-20

**Authors:** Chiaki Murakami, Fumio Sakane

**Affiliations:** Department of Chemistry, Graduate School of Science, Chiba University, Chiba, Japan

**Keywords:** sphingomyelin synthase-related protein, phosphatidic acid phosphatase, phosphatidylinositol phospholipase C, phosphatidylethanolamine phospholipase C, diacylglycerol kinase, Cer, ceramide, CHS, cholesteryl hemisuccinate, CPE, ceramide phosphoethanolamine, DDM, n-dodecyl-β-D-maltoside, DG, diacylglycerol, DGK, diacylglycerol kinase, ER, endoplasmic reticulum, I.S., internal standard, LPP, lipid phosphate phosphatase, MG-PLC, multi-glycerophospholipid PLC hydrolase, PA, phosphatidic acid, PAP, phospholipid phosphatase, PC, phosphatidylcholine, PE, phosphatidylethanolamine, PG, phosphatidylglycerol, PI, phosphatidylinositol, PLC, phospholipase C, PMSF, phenylmethylsulfonyl fluoride, SM, sphingomyelin, SMS, sphingomyelin synthase, SMSr, sphingomyelin synthase-related protein

## Abstract

Diacylglycerol (DG) is a well-established lipid second messenger. Sphingomyelin synthase (SMS)-related protein (SMSr) produces DG and ceramide phosphoethanolamine (CPE) by the transfer of phosphoethanolamine from phosphatidylethanolamine (PE) to ceramide. We previously reported that human SMSr overexpressed in COS-7 cells significantly increased DG levels, particularly saturated and/or monounsaturated fatty acid–containing DG molecular species, and provided DG to DG kinase (DGK) δ, which regulates various pathophysiological events, including epidermal growth factor–dependent cell proliferation, type 2 diabetes, and obsessive–compulsive disorder. However, mammalian SMSr puzzlingly produces only trace amounts of CPE/DG. To clarify this discrepancy, we highly purified SMSr and examined its activities other than CPE synthase. Intriguingly, purified SMSr showed a DG-generating activity *via* hydrolysis of PE, phosphatidic acid (PA), phosphatidylinositol (PI), and phosphatidylcholine (PC) in the absence of ceramide. DG generation through the PA phosphatase (PAP) activity of SMSr was approximately 300-fold higher than that with PE and ceramide. SMSr hydrolyzed PI ten times stronger than PI(4,5)bisphosphate (PI(4,5)P_2_). The PAP and PC-phospholipase C (PLC) activities of SMSr were inhibited by propranolol, a PAP inhibitor, and by D609, an SMS/PC-PLC inhibitor. Moreover, SMSr showed substrate selectivity for saturated and/or monounsaturated fatty acid–containing PA molecular species, but not arachidonic-acid-containing PA, which is exclusively generated in the PI(4,5)P_2_ cycle. We confirmed that SMSr expressed in COS-7 cells showed PAP and PI-PLC activities. Taken together, our study indicated that SMSr possesses previously unrecognized enzyme activities, PAP and PI/PE/PC-PLC, and constitutes a novel DG/PA signaling pathway together with DGKδ, which is independent of the PI(4,5)P_2_ cycle.

Diacylglycerol (DG) is a well-established lipid second messenger that activates protein kinase C (PKC) (1, 2). In addition to PKC, DG regulates a wide variety of signal transduction proteins, such as β2-chimaerin, protein kinase D, Ras guanyl nucleotide-releasing protein, and Unc-13 (3, 4, 5, 6). In eukaryotes, DG is generated from two major lipids: the glycerolipids, such as monoacylglycerol and triacylglycerol (7, 8, 9) *via* acyltransferase and lipase, respectively, and glycerophospholipids, such as phosphatidic acid (PA), phosphatidylinositol 4,5-bisphosphate (PI(4,5)P_2_), phosphatidylcholine (PC), and phosphatidylethanolamine (PE) *via* PA phosphatase (PAP)/lipid phosphate phosphatase (LPP) or phospholipase C (PLC) (10, 11, 12, 13). Although type II PAP/lipid phosphate phosphatase (PAP2/LPP), lipin (type I PAP) and PI(4,5)P_2_-specific phospholipase C (PLC), as DG-generating enzymes, have been cloned and extensively studied in mammals (10, 14, 15), the molecular entities (the genes and proteins) of PC- and PE-specific PLCs have not been identified until now (12, 16).

Sphingomyelin synthase (SMS) is a DG-generating enzyme (17, 18). There are three isoforms, SMS1, SMS2, and SMS-related protein (SMSr) (Table 1). Huitema *et al.* (19) identified SMS genes *via* a homology search for the sequences encoding the membrane proteins containing active site motifs common to PAP2/LPP. The SMS1 and SMS2 proteins generate DG and SM *via* the transfer of phosphocholine from PC to ceramide (Table 1). SMSr is an endoplasmic reticulum (ER)-resident, six-transmembrane protein and has no SMS activity but displays ceramide phosphoethanolamine (CPE) synthase (CPES) activity *via* the transfer of phosphoethanolamine from PE to ceramide (20) (Table 1). Puzzlingly, mammalian SMSr produces only trace amounts of CPE (approximately 300-fold lower than SM levels produced by SMS1) (20) (Table 1). Moreover, CPE levels in mammalian cells are extremely low (0.002%–0.005 mol% of total phospholipids) (21). Hence, it is supposed that SMSr has little or no ceramide-dependent DG-generating activity.

**Table 1:** Comparison of SMSr with other SMSs

Properties		SMSr (current study)	SMSr (previous reports)	SMS1	SMS2
Subcellular localization			ER (active site: lumen) (20, 47)	Golgi (19, 73, 74)	Plasma membrane, Golgi (19, 73)
Reaction	Substrates	1. PA 2. PI>PIP_2_ (selectivity 10:1) 3. PE 4. PC 5. PG	Cer + PE (20) (approximately 300-fold lower than SM levels produced by SMS1)	1. Cer + PC (19, 74) 2. Cer + PE (75)	1. Cer + PC (19) 2. Cer + PE (47, 75)
	Products (Lipids)	DG	CPE + DG	1. SM, DG 2. CPE, DG	1. SM, DG 2. CPE, DG
Inhibitor	Propranolol	PAP activity PC-PLC activity	Unknown	Unknown	Unknown
	D609	PAP activity PC-PLC activity	Unknown	SMS activity (40, 76, 77)	SMS activity (76, 77)
	Others (SM synthesis inhibitor)			Marabaricone C (78) SAPA (79)	α-aminonitrile (compound D2) (80) 2-quinolone (77) 2-benzyloxybenzamides (Ly93) (81) Marabaricone C (78)

Recently, we demonstrated that SMSr interacted with the δ isozyme of DG kinase (DGKδ) (22, 23, 24), which is involved in the pathogenesis of type 2 diabetes (25, 26), obsessive–compulsive disorder (27, 28), and epidermal growth factor–dependent cell proliferation (29), *via* their sterile α motif domains (SAMDs) (30). Moreover, overexpression of both SMSr and DGKδ, but not of SMSr or DGKδ alone, enhanced PA production in COS-7 cells (30). In particular, the levels of saturated fatty acid (SFA)- and/or monounsaturated fatty acid (MUFA)-containing PA species, including 16:1/16:1-PA, 16:0/16:1-PA, 16:0/16:0-PA, 16:1/18:1-PA, and 16:0/18:1-PA, which were also produced by DGKδ in high-glucose-stimulated C2C12 myoblast cells (31), were significantly increased. Furthermore, SMSr overexpressed in COS-7 cells generated SFA- and/or MUFA-containing DG species. Therefore, it is possible that SMSr acts upstream of DGKδ and provides SFA- and/or MUFA-containing DG.

Taken together, SMSr can supply DG to DGKδ (31), whereas SMSr has little or no ceramide-dependent DG-generating activity (CPES activity) (20, 21). These contradictory results prompted us to postulate that SMSr possesses other DG-generating activity in addition to CPES activity. Here, we report that human SMSr, which was expressed in Sf9 cells and is highly purified, displayed DG-generating activity *via* hydrolysis of PA, PI, PE, and PC in the absence of ceramide. The PAP, PI-phospholipase C (PLC), PE-PLC, and PC-PLC activities were much stronger than the CPES activity (∼300-fold, ∼10-fold, ∼4-fold, and ∼3-fold, respectively). Intriguingly, SMSr exhibited a substrate specificity for SFA- and/or MUFA-containing PA and PC molecular species, but not polyunsaturated fatty acids (PUFA)-containing PA and PC. Taken together, we revealed the previously unrecognized enzyme activities of mammalian SMSr, novel PAP, PI-PLC, PE-PLC, and PC-PLC activities, to produce SFA and/or MUFA-containing DG molecular species independent of ceramide in the ER membrane.

## Results

### Purification of human SMSr

**Figure 1:**
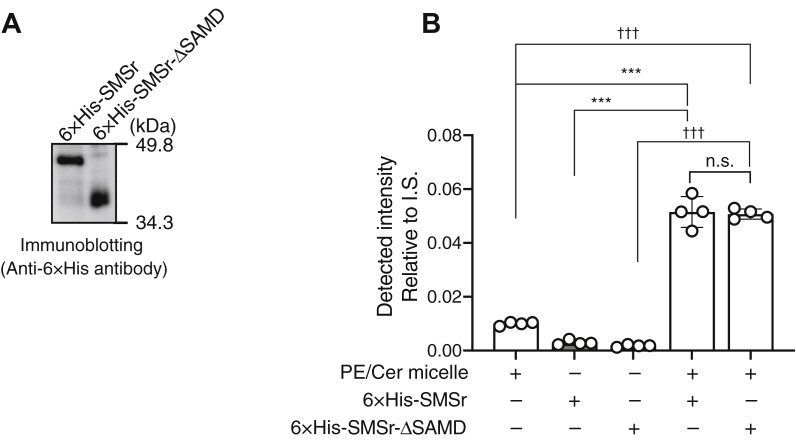
**DG-generating activity of partially purified SMSr in the presence of PE and ceramide.***A*, partial purification of 6× His-SMSr and 6× His-SMSr-ΔSAMD expressed in Sf9 cells only *via* Ni-NTA agarose affinity chromatography. Partially purified 6× His-tagged proteins were detected *via* immunoblotting using anti-6× His antibody (PM032, Medical & Biological Laboratories, 1:1000 dilution). *B*, DG-generating activities of 6× His-tagged SMSr and SMSr-ΔSAMD were measured using LC-MS/MS. Partially purified 6× His-tagged proteins only *via* Ni-NTA agarose affinity chromatography (100 μl of sample) were used for the enzyme assay. The substrates (100 μl of 1 mM d18:1/18:0-ceramide, 1 mM 16:0/18:1-PE and 50 mM octyl-β-D-glucoside mixed-micelles) were used for the enzyme activity assay. The values are presented as the mean ±S.D. (n = 3). ∗∗∗*p* < 0.005 (*versus* 6× His-SMSr), ^†††^*p* < 0.005 (*versus* 6× His-SMSr-ΔSAMD), n.s., not significant. PE and Cer mixed micelles contained detectable 16:0/18:1-DG (0.547 pmol/sample) that probably came from a PE stock solution. However, it was negligible because contamination rate was 0.0005%.

To determine whether SMSr possesses other DG-generating activity in addition to CPES activity, we first expressed N-terminal hexahistidine (6×His)-tagged human full-length (FL) SMSr and SMSr-ΔSAMD, which lacks an oligomer formation domain, SAMD (32, 33), in Sf9 cells and purified them from Sf9 cell membranes using Ni-affinity chromatography (Fig. 1*A*). SMSr-ΔSAMD showed no significant difference in DG production levels compared with FL-SMSr (Fig. 1*B*). However, the SMSr-ΔSAMD yield was approximately six-fold higher than that of FL-SMSr (data not shown). Therefore, we employed SMSr-ΔSAMD for further investigation.

**Figure 2:**
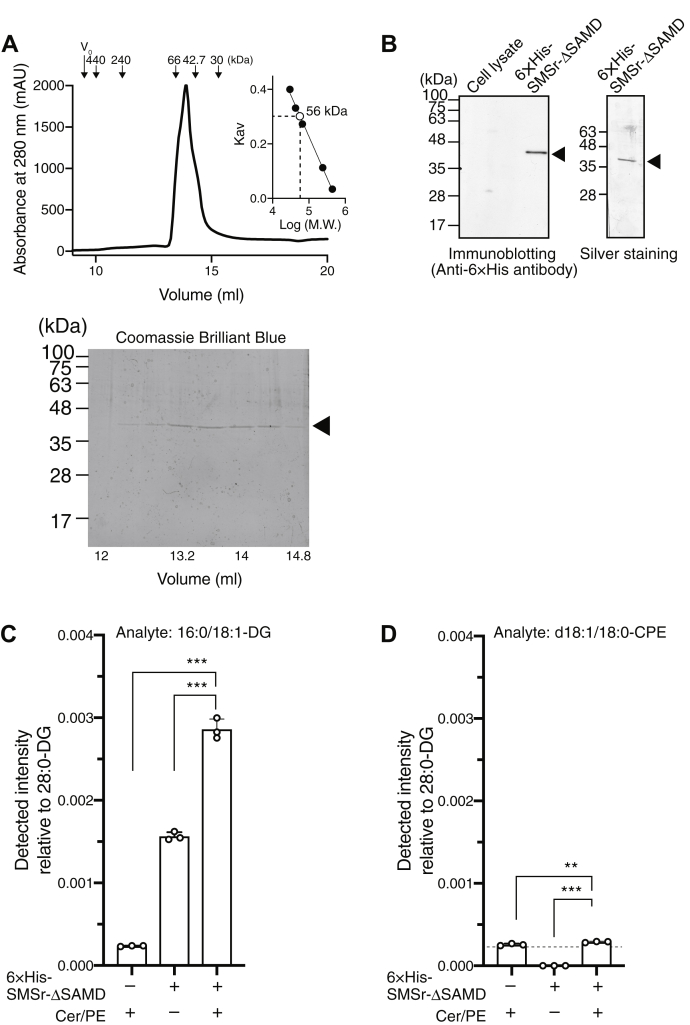
**Purification of SMSr-ΔSAMD.***A*, elution profile of 6× His-SMSr-ΔSAMD from size-exclusion chromatography. The *inset* shows the calibration curve constructed using ferritin (440 kDa), catalase (240 kDa), bovine serum albumin (66 kDa), ovalbumin (42.7 kDa), and carbonic anhydrase (30 kDa). V_0_ is the void volume of the column (9.52 ml). The fractions corresponding to the peaks of SMSr-ΔSAMD were collected and concentrated. The separated proteins were detected by an SDS-PAGE (12%) analysis followed by Coomassie blue staining. *B*, purified 6× His-SMSr-ΔSAMD was analyzed *via* SDS–polyacrylamide gel electrophoresis (12% gel) followed by silver staining or immunoblotting using anti-6× His antibody (PM032, Medical & Biological Laboratories). *C*, the DG-generating activities of purified SMSr-ΔSAMD were measured using LC-MS/MS. d18:1/18:0-Cer and 16:0/18:1-PE micelles were used as substrates. The detected intensity of 16:0/18:1-DG in the sample was quantified using an internal standard (I.S.) (0.5 ng/μl of 14:0/14:0-DG). The values are presented as the mean ± S.D. (n = 3). Purified SMSr-ΔSAMD contained detectable 16:0/18:1-DG that probably came from Sf9 cell membranes. We calculated DG-generating activity of SMSr-ΔSAMD after subtracting these backgrounds. *D*, the CPES activity of SMSr-ΔSAMD was measured using LC-MS/MS. d18:1/18:0-Cer and 16:0/18:1-PE micelles were used as substrates. In positive ion mode, d18:1/18:0-CPE [M–H]^+^ (*m/z* 689.6) was selected as the precursor ion (Q1). The long-chain base (*m/z* 300.3) generated by CID of *m/z* 689.6 was selected as the product ion (Q3). The detected intensity of CPE in the sample was quantified using 0.5 ng/μl of 1,2-dimyristoyl-sn-glycerol (28:0-DG). The values are presented as the mean ±S.D. (n = 3).

Next, to further purify 6× His-SMSr-ΔSAMD, size-exclusion chromatography was conducted after Ni-affinity chromatography. SMSr-ΔSAMD was eluted as a single peak in a volume of 13.9 ml (∼56.1 kDa) (Fig. 2*A*). Thus, the purified 6× His-SMSr-ΔSAMD is likely to be a monomer because its calculated molecular mass is 40.8 kDa (SMSr-ΔSAMD: 39.2 kDa +6×His: 1.6 kDa). Immunoblotting and silver staining showed that a single protein band at approximately 40 kDa was detected (Fig. 2*B*), indicating that 6× His-SMSr-ΔSAMD was obtained with a high purity. The yield was ∼6 μg per 1 L of an Sf9 cell culture. We confirmed that the purified SMSr-ΔSAMD slightly produced DG in the presence of PE and ceramide micelles (Fig. 2*C*). However, the DG-generating activity was exceedingly low (∼3 pmol/mg/min). Moreover, only trace amounts of CPE were generated by the purified SMSr-ΔSAMD (Fig. 2*D*).

### Enzymological characterization of SMSr activity

**Figure 3:**
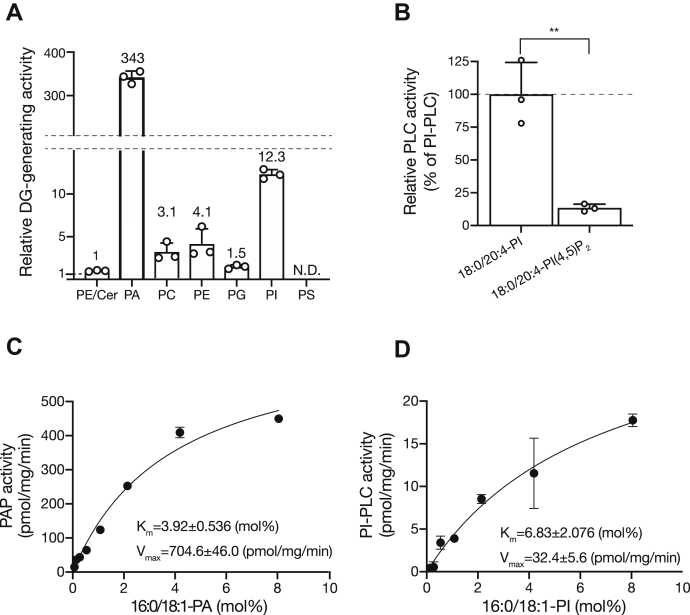
**Enzymological characterization of SMSr-ΔSAMD activity.***A*, purified 6× His-SMSr-ΔSAMD (10 μl (0.03 μg/μl) of sample) was used for the enzyme assay using N-Stearoyl-D-Sphingosine (d18:1/18:0-Cer) and 1-palmitoyl-2-oleoyl (16:0/18:1)-PE mixed micelles (Cer/PE), 16:0/18:1-PA, 16:0/18:1-PC, 16:0/18:1-PE, 16:0/18:1-PG, 16:0/18:1-PI, and 16:0/18:1-PS, as substrates. Relative DG-generating activities were compared with that in the presence of Cer/PE (0.8653 pmol/mg/min) (set to 1). The results are presented as the mean ± S.D. (n = 3). *B*, comparison of the enzyme activities for SMSr-ΔSAMD with 18:0/20:4-PI and 18:0/20:4-PI(4,5)P_2_ as substrates. The PLC activities of SMSr-ΔSAMD were measured using LC-MS/MS. The relative PIP_2_-PLC activity was standardized by comparison with the PI-PLC activity (7.97 pmol/mg/min) (set to 100%). The values are presented as the mean ± S.D. (n = 3). ∗∗*p* < 0.01. *C* and *D*, enzyme kinetic analyses of purified SMSr with (*C*) 16:0/18:1-PA and (*D*) 16:0/18:1-PI. The DG-generating activities were plotted as a function of (*C*) 16:0/18:1-PA concentration and (*D*) 16:0/18:1-PI concentration in mixed micelle (mol%). The values are the averages of triplicate determinations. The data are represented as the mean ± S.D.

To test whether SMSr-ΔSAMD hydrolyzes other phospholipids, we compared the hydrolysis activity of 10 μl (0.03 μg/μl) of purified SMSr-ΔSAMD against various phospholipids (1-palmitoyl-2-oleoyl (16:0/18:1)-PA, 16:0/18:1-PC, 16:0/18:1-PE, 16:0/18:1-PG, 16:0/18:1-PI and 16:0/18:1-PS) *in vitro* by incubating the substrate micelle mixture followed by quantitation of the produced DG levels using liquid chromatography–tandem mass spectrometry (LC-MS/MS) (30, 34) (Fig. 3*A*). Compared with the DG-generating activity in the presence of ceramide and 16:0/18:1-PE (substrates for CPES), SMSr showed a much more intensive PAP activity (∼340-fold), even in the absence of ceramide. The enzyme also has stronger PI-PLC (∼12-fold), PE-PLC (∼4-fold), PC-PLC (∼3-fold), and PG-PLC (∼1.5-fold) activities. However, the PS hydrolysis activity of SMSr was not detectable. To further investigate the selectivity of SMSr for phosphoinositides, we compared the hydrolysis activities of purified SMSr-ΔSAMD against 18:0/20:4-PI and 18:0/20:4-PI(4,5)P_2_ (Fig. 3*B*). The enzyme showed a ten-fold stronger PI-PLC activity than the PI(4,5)P_2_-PLC activity. This result indicates that the catalytic selectivity for the phosphoinositides of SMSr is opposite to that of mammalian PI-PLC (PI(4,5)P_2_-PLC), which hydrolyzes PI(4,5)P_2_ much more strongly than PI (PI(4,5)P_2_:PI = 50:1) (Table 2) (35).

**Table 2:** Comparison of SMSr with other DG-generating enzymes

Properties		SMSr (current study)	SMSr (previous reports)	LPP (PAP2)	Lipin-1	PI(4,5)P_2_-PLC	PE-PLC	PC-PLC
Subcellular localization			ER (active site: lumen) (20, 47)	PM>>ER, Golgi, endosome (14, 42, 51)	Cytosol or nucleus (82)	Cytosol (10, 35)	Unknown	Membrane bound? (83, 84, 85)
Reaction	Substrates	1. PA 2. PI>PIP_2_ (selectivity 10:1) 3. PE 4. PC 5. PG	Cer + PE	1. PA 2. LPA 3. S1P 4. C1P	PA	PI(4,5)P_2_>PI (selectivity 50:1) (54)	PE	PC
	Products (Lipids)	DG	CPE + DG (20)	1. DG 2. MG 3. Sphingosine 4. Cer	DG	DG	DG	DG
Inhibitor	Propranolol	PAP activity PC-PLC activity	Unknown	PAP: inhibit (slightly) (36, 71)	inhibit (strongly) (37)	Unknown	Unknown	Unknown
	D609	PAP activity PC-PLC activity	Unknown	Unknown	Unknown	Unknown	Inhibit (∼100 μM) (13)	Inhibit (∼100 μM) (40, 72)
Kinetic parameter (*K*_m_)		16:0/18:1-PA: 3.92 ± 0.536 mol% (97.4 ± 13.3 μM) 16:0/18:1-PI: 6.83 ± 2.08 mol% (169.7 ± 51.7 μM)	Unknown	PA (36) LPP1: 3.4 ± 0.05 mol% LPP2: 0.44 ± 0.07 mol% LPP3: 0.61 ± 0.03 mol%	PA (37) Lipin-1α: 4.2 ± 0.11 mol% Lipin-1β: 4.5 ± 0.07 mol% Lipin-1γ: 4.3 ± 0.14 mol%	PLCδ1 (86) PI: 130 μM PI(4,5)P_2_: 170 μM	Unknown	Unknown

These results suggested that SMSr not only acted as CPES but also a DG-generating enzyme through PAP, PI-PLC, PE-PLC, PC-PLC, PIP_2_-PLC, and PG-PLC activities. Next, a kinetic analysis of the enzyme was performed using n-dodecyl-β-D-maltoside (DDM)/Cholesteryl hemisuccinate (CHS)/phospholipid-mixed micelles. The *K*_m_ value for 16:0/18:1-PA was 3.92 ± 0.536 mol% (*V*_max_: 704.6 ± 46 pmol/mg/min) (Fig. 3*C*), and the value for 16:0/18:1-PI was 6.83 ± 2.076 (mol%) (*V*_max_: 32.4 ± 5.6 pmol/mg/min) (Fig. 3*D*). The *K*_m_ value and *V*_max_ for 16:0/18:1-PA are comparable with those of LPPs (36) and lipin-1s (37) (Table 2).

**Figure 4:**
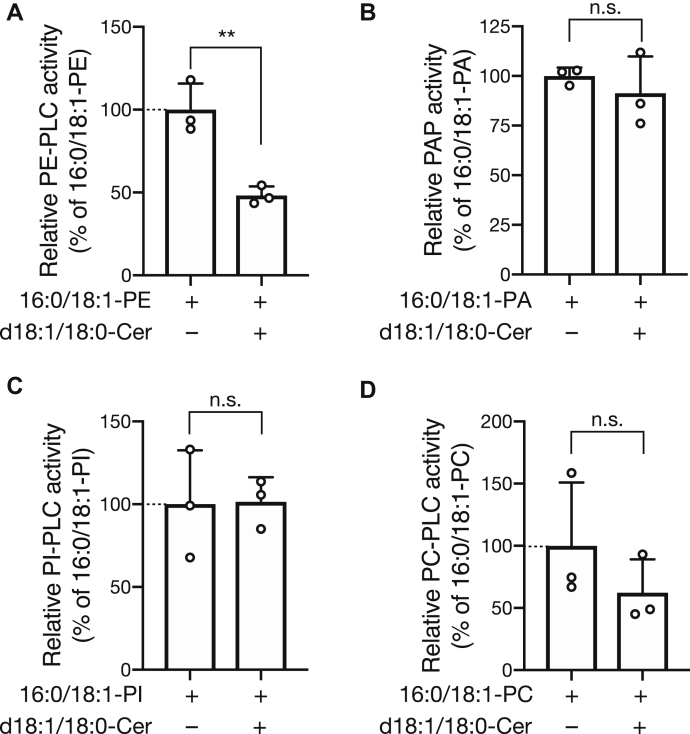
**Effect of d18:1/18:0-ceramide on the DG-generating activity of SMSr-ΔSAMD.** PE-PLC (*A*), PAP (*B*), PI-PLC (*C*), and PC-PLC (*D*) activities of the SMSr-ΔSAMD were analyzed in the presence or absence of 50 μM (2.1 mol%) d18:1/18:0-Cer, which is a substrate of SMSr as a CPE synthase (20). The values are presented as percentages of the activity of SMSr-ΔSAMD in the absence of d18:1/18:0-Cer (−) (16:0/18:1-PE-PLC, 7.42 pmol/mg/min;16:0/18:1-PAP, 194.7 pmol/mg/min; 16:0/18:1-PI-PLC 15.0 pmol/mg/min; 16:0/18:1-PC-PLC, 8.13 pmol/mg/min) (set to 100%). The values are presented as the mean ± S.D. (n = 3). ∗∗*p* < 0.01; n.s., not significant.

Because DG production activity by SMSr-ΔSAMD in the presence of d18:1/18:0-ceramide and PE was lower than that in presence of PE alone (Fig. 3*A*), we assumed that ceramide affects DG-generating activity of SMSr-ΔSAMD. Thus, the effects of d18:1/18:0-ceramide on hydrolysis activities of SMSr-ΔSAMD for other glycerophospholipids (PA, PI, and PC) were determined (Fig. 4). We confirmed that PE-PLC activity of SMSr-ΔSAMD was significantly decreased in presence of d18:1/18:0-ceramide (Fig. 4*A*). However, ceramide had no detectable effects on hydrolysis of PA, PI, and PC (Fig. 4, *B*–*D*), suggesting that the inhibitory effect of ceramide is PE selective.

**Figure 5:**
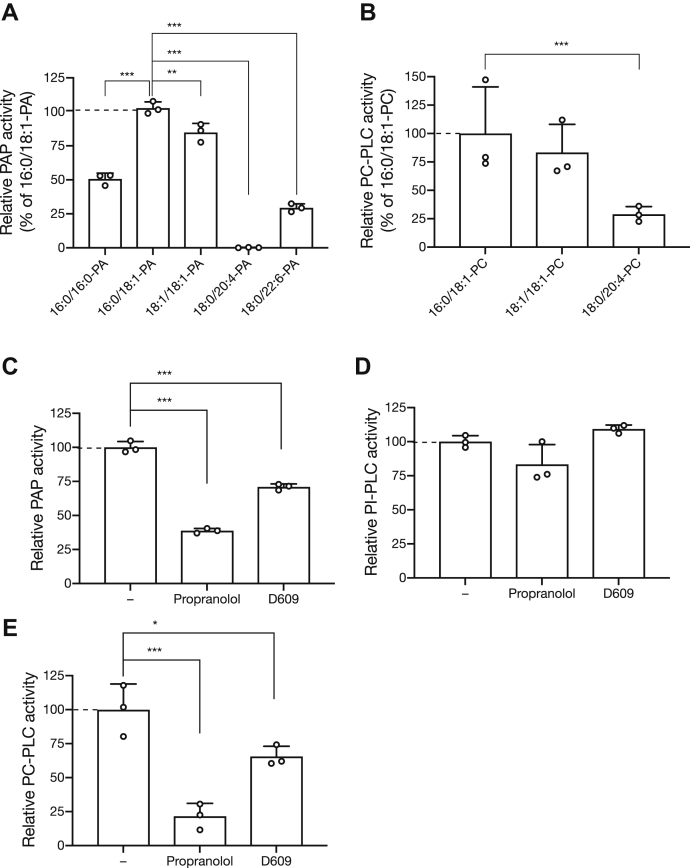
**Effects of acyl chains of phospholipids and inhibitors of PAP and SMS/PC-PLC on the SMSr-ΔSAMD activity.***A*, comparison of the enzyme activities for SMSr-ΔSAMD with 16:0/16:0-, 16:0/18:1-, 18:1/18:1-, 18:0/20:4-, and 18:0/22:6-PA as substrates. The detected intensity of the DG molecular species using LC-MS/MS was converted into the amount of DG produced (pmol) based on the calibration curves measured separately with a known concentration of each DG species. The relative PAP activity levels were standardized by comparison with the 16:0/18:1-PAP activity (505.2 pmol/mg/min) (set to 100%). *B*, comparison of the enzyme activities for SMSr-ΔSAMD with 16:0/18:1-, 18:1/18:1-, 18:0/20:4-PC as substrates. The relative PC-PLC activity was standardized by comparison with the 16:0/18:1-PC-PLC activity (2.75 pmol/mg/min) (set to 100%). *C*–*E*, PAP (*C*), PI-PLC (*D*) and PC-PLC (E) activities of the SMSr-ΔSAMD were analyzed in the presence of 1 mM propranolol, which is a PAP inhibitor (36, 71), or 500 μM D609, which is a PC-PLC and SMS inhibitor (12, 40, 72). The values are presented as percentages of the activity of SMSr-ΔSAMD in the absence of the inhibitor (−) (16:0/18:1-PAP, 817.1 pmol/mg/min; 16:0/18:1-PI-PLC, 29.3 pmol/mg/min; 16:0/18:1-PC-PLC, 2.66 pmol/mg/min) (set to 100%). The values are presented as the mean ± S.D. (n = 3). ∗*p* < 0.05; ∗∗∗*p* < 0.005 (*versus* control (−)).

The substrate selectivity of SMSr for PA and PC having different acyl chains was examined (Fig. 5, *A* and *B*). SMSr was intensively active against SFA and/or MUFA-containing PA species, such as 16:0/18:1-, 16:0/16:0-, and 18:1/18:1-PA. However, SMSr exhibited relatively low activities against PUFA-containing PA, such as 18:0/20:4-PA, which is exclusively derived from the PI(4,5)P_2_ cycle (38), and 18:0/22:6-PA.

We examined the effects of propranolol, which is a well-known PAP inhibitor (36, 39), and D609, which is a SMS and PC-PLC inhibitor (40,41) (Tables 1 and 2), on PAP, PI-PLC, and PC-PLC activities of SMSr (Fig. 5, *C*–*E*). The PAP and PC-PLC activities were significantly inhibited by 1 mM propranolol (approximately 60% and 80% decreases, respectively) and by 500 μM D609 (approximately 30% and 20% decreases, respectively) (Fig. 5, *C* and *E*). However, the inhibitors had no significant effects on the PI-PLC activity (Fig. 5*D*).

**Figure 6:**
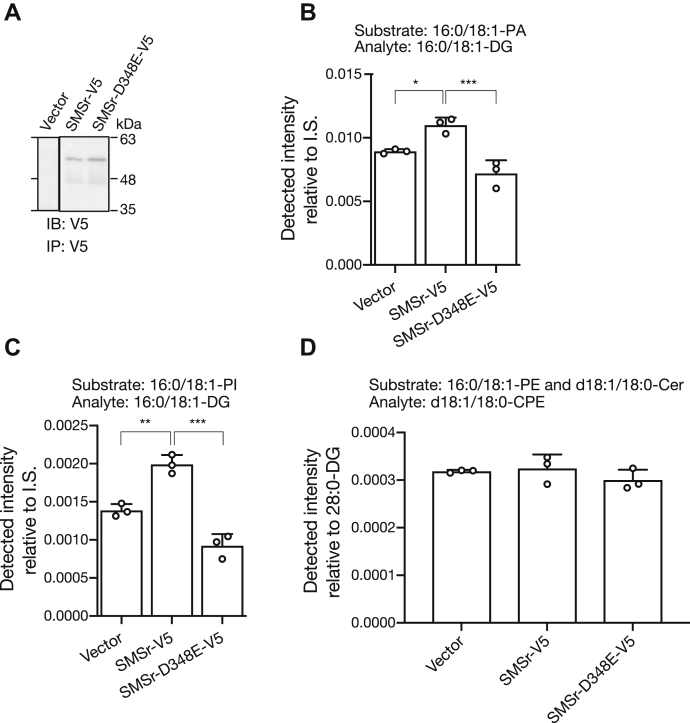
**PAP activity of SMSr expressed in mammalian cells.***A*, SMSr-V5 and SMSr-D348E were overexpressed in COS-7 cells. After 24 h of transfection, the cells were lysed and immunoprecipitated with an anti-V5 antibody (MA5-15253, Invitrogen), followed by immunoblotting to confirm the expression of SMSr-V5 using the anti-V5 antibody. *B*, PAP, *C*, PI-PLC, and *D*, CPES activities in immunoprecipitates were measured by quantitation of 16:0/18:1-DG or d18:1/18:0-CPE levels using LC-MS/MS. The values are presented as the mean ± S.D. (n = 3). ∗*p* < 0.05 (*versus* SMSr-V5).

To test whether human SMSr expressed in mammalian cells, not insect cells, also displayed PAP and PI-PLC activities, C-terminal V5-tagged SMSr was expressed in COS-7 cells. The V5-tagged SMSr was immunoprecipitated using an anti-V5 tag antibody (Fig. 6*A*), and PAP, PI-PLC, and CPES activities in the immunoprecipitates were measured (Fig. 6, *B*–*D*). The SMSr expressed in the COS-7 cells exhibited PAP and PI-PLC activities (Fig. 6, *B* and *C*). However, a CPE synthase-inactive mutant of SMSr (D348E) (20) failed to show PAP and PI-PLC activities, indicating that Asp348 is also important for PAP and PI-PLC activities. On the other hand, the CPES activity was too low to detect in the precipitates (Fig. 6*D*), suggesting that human SMSr lacks the ability to synthesize considerable amounts of CPE, as Vacaru *et al.* reported (20).

**Figure 7:**
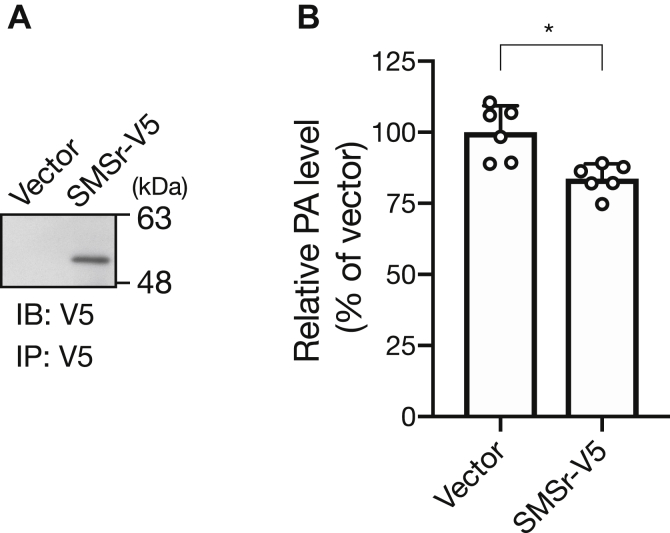
**Changes in the amounts of total PA in COS-7 cells by overexpressing SMSr.***A*, human SMSr-V5 was overexpressed in COS-7 cells. After 24 h of transfection, the cells were lysed and immunoprecipitated with an anti-V5 antibody (MA5-15253, Invitrogen), followed by immunoblotting to confirm the expression of SMSr-V5 using the anti-V5 antibody. *B*, total PA levels in COS-7 cells transfected with vector alone or SMSr-V5 were measured using LC-MS/MS (30). The values are presented as the mean ± S.D. (n = 6). ∗*p* < 0.05.

We previously reported that SMSr expressed in COS-7 cells generated DG (30). To determine whether SMSr indeed utilizes PA to produce DG in mammalian cells, we analyzed the changes in the amounts of PA in COS-7 cells overexpressing SMSr-V5 (Fig. 7*A*). The total PA levels in COS-7 cells overexpressing SMSr-V5 were significantly decreased (Fig. 7*B*). This result suggests that SMSr produced DG, at least in part, by hydrolyzing PA in mammalian cells.

## Discussion

**Figure 8:**
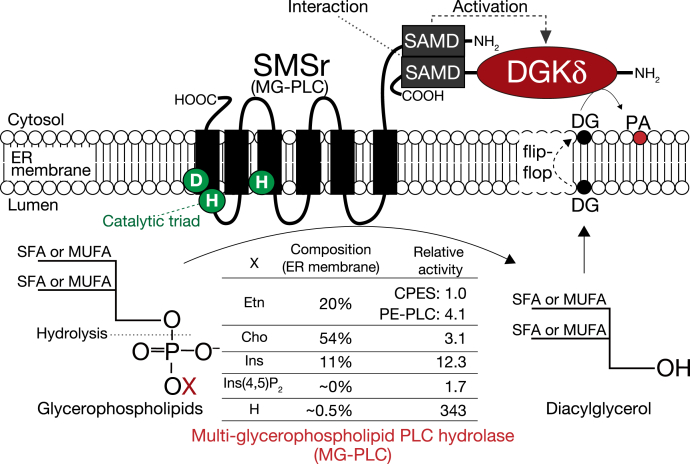
**Model for a new DG-signaling pathway involving SMSr and DGKδ.** In the present study, we demonstrated that SMSr displayed PAP, PI-PLC, PE-PLC, PC-PLC, and PG-PLC activities that were stronger than the CPE synthase activity (20). Moreover, the SMSr exhibited a substrate specificity for SFA- and/or MUFA-containing PA and PC molecular species, but not PUFA-containing PA and PC. It is possible that SMSr preferably hydrolyzes SFA- and/or MUFA-containing glycerophospholipids independent of the ceramide. Previously, we reported that SMSr and DGKδ functionally interact *via* their SAMDs (30). Taken together, it is possible that there is a new mammalian DG-signaling pathway consisting of SMSr and DGKδ independent of the PI(4,5)P_2_ cycle.

In the present study, we answered a major question: why does SMSr only exhibit negligible CPES activity? We found that SMSr is a new type of enzyme that is able to hydrolyze multiple glycerophospholipids, such as PA, PI, PE, and PC, to produce DG (Fig. 8). SMSr is a novel PAP that primarily acts in the ER lumen unlike the previously reported PAP/LPP (42, 43). Intriguingly, SMSr is true mammalian PI-PLC, while the previously found so-called PI-PLC is PIP_2_-PLC (10, 15, 35). Moreover, our data indicate that SMSr is a strong candidate for the elusive mammalian PE-PLC and PC-PLC activities, which were first described about 40 years ago (44, 45, 46). Furthermore, we answered another question: why does DGKδ, which does not show selectivity to DG species *in vitro* (31), apparently utilize limited DG species, 16:0 and/or 16:1-containing DG. The intrinsic selectivity of SMSr, which functions upstream of DGKδ, for 16:0 and/or 16:1-containing substrates would determine the limitation of DG species utilized by DGKδ.

The purification of six-transmembrane proteins, SMS and SMSr, is generally difficult (18). However, in order to analyze enzymatic activities of SMSr beyond its previously reported CPES activity, purification was first needed. Thus, we expressed human SMSr using the baculovirus-insect cell system and, through trial and error, highly purified it *via* affinity and size-exclusion chromatography (Fig. 2, *A* and *B*). The purified enzyme gave almost a single band with a molecular mass of 43,000 *via* SDS gel electrophoresis. It is likely that size-exclusion chromatography with a buffer containing a nonionic, nondenaturing detergent, Nonidet P-40 (NP-40), at critical micelle concentration (0.018%) was critical for the successful purification.

Using the highly purified enzyme, we demonstrated for the first time that SMSr displayed not only CPES activity but also PAP, PE-PLC, PI-PLC, PC-PLC, and PG-PLC activities *in vitro* (Fig. 3, *A* and *B*). Interestingly, compared with the DG-generating activity in the presence of PE and ceramide (substrates of CPE synthase), purified human SMSr showed a ∼340-fold stronger PAP activity, even in the absence of ceramide. Considering the report that SMSr metabolized approximately 300-fold less ceramide than SMS1 (20) (Table 1), it is likely that the PAP activity of human SMSr is comparable with the SM synthase activity of SMS1. Moreover, we determined the *K*_m_ values for 16:0/18:1-PA and 16:0/18:1-PI of human SMSr (3.92 and 6.83 mol%) (Fig. 3, *C* and *D*), which are almost the same as those of other PAPs, LPP1 (PAP2a) (36), and lipin-1 (37) (Table 2). We demonstrated that Asp348 of SMSr is part of the enzyme’s active site, which faces the ER lumen (19, 20, 47), was important for not only for CPES, but also for PAP and PI-PLC activities (Fig. 6, *B* and *C*). Therefore, SMSr probably shows CPES, PAP, PE-PLC, PI-PLC, PC-PLC, and PG-PLC activities through the same catalytic site.

In this study, we found that ceramide selectively affected PE-PLC activity of SMSr-ΔSAMD (Fig. 4*A*), but not its PAP, PI-PLC, or PC-PLC activity (Fig. 4, *B*–*D*). It is possible that PE-PLC activity of SMSr is competitive with CPE synthase activity of SMSr. SMSr has CPE synthase activity and, consequently, ceramide can occupy the substrate (ceramide/PE) binding site of SMSr (20). Therefore, it is likely that d18:1/18:0-ceramide interferes with PE-PLC activity by blocking access of PE to the active site. It was reported that mammalian SMSr acts as not only CPE synthase, but also ceramide sensor in the ER to protect cells against ceramide-induced mitochondrial apoptosis (20, 48). Taken together, SMSr modulates ceramide levels in ER *via* its SAMD, and vice versa, ceramide may selectively modulate the function (PE-PLC activity) of SMSr at least in part.

The PI-PLC, PE-PLC, and PC-PLC activities of purified SMSr were 28∼100-fold weaker than the PAP activity (Fig. 3*A*). However, two lines of evidence support that the PI-PLC activity of SMSr is comparable with its PAP activity in cells. First, the amount of PI, PC, and PE in the ER membrane was more than 20-fold, 100-fold, and 40-fold higher than that of PA, respectively (11%, 54%, and 20% *versus* less than 0.5% of total lipids in the ER membrane, respectively) (Fig. 8) (49, 50). As shown in Figure 3, *C* and *D*, SMSr showed a PAP activity of ∼60 pmol/mg/min at 0.5 mol% PA and ∼18 pmol/mg/min at 8 mol% PI. Second, the SMSr expressed in mammalian cells showed a PI-PLC activity as strong as (only 3.5-fold lower) the PAP activity (Fig. 5, *B* and *C*). These results suggest that the PI-PLC activity of SMSr expressed in mammalian cells is comparable with its PAP activity. Therefore, PAP and PI-PLC (and PE-PLC and PC-PLC) are likely to represent the substantial activity of SMSr.

What are the differences between SMSr as PAP and the already-identified PAPs (PAP2/LPP and lipin-1) (see Table 2)? LPPs have broad substrate specificity (see Table 2). However, they do not hydrolyze PC, PE, PI, or phosphoinositide (PI(4,5)P_2_) (11, 14, 51). Interestingly, SMSr hydrolyzed PA, PI, PI(4,5)P_2_, PE, PG, and PC (Fig. 3, *A* and *B*). LPPs contain six transmembrane domains and are primarily localized to the plasma membrane (14, 42, 51). It was reported that LPP2 and LPP3 were only partly localized to the ER in addition to the plasma membrane (52, 53). SMSr is an exclusive ER membrane protein (20, 33). Asp348 of SMSr is one of the catalytic triad on the ER luminal side (Fig. 8). It is possible that SMSr as PAP hydrolyzes PA and PI/PC/PE in the ER lumen, in contrast to other PAPs (LPPs and lipins, which are cytosolic proteins).

Lipin-1, the most characterized type I PAP, is specific for PA. Lipin-1 hydrolyzed 1,2-diunsaturated and 1-saturated-2-unsaturated PA molecular species but hydrolyzed little or no saturated PA (16:0/16:0- and 18:0/18:0-PA) (37). On the other hand, SMSr selectively hydrolyzed SFA and/or MUFA-containing PA and PC molecular species, but not 20:4-containing PA and PC (Fig. 4, *A* and *B*). This unique selectivity reinforces the hypothesis that SMSr and its downstream enzyme, DGKδ, are components of the alternative DG/PA-signaling pathway, which is independent of the PIP_2_ cycle (Fig. 8).

What are the differences between SMSr as PI-PLC and PI(4,5)P_2_-PLC? We found that SMSr also hydrolyzed 18:0/20:4-PI(4,5)P_2_ *in vitro*, and this activity was approximately tenfold weaker than the 18:0/20:4-PI-PLC activity (Fig. 3*B*). Notably, although mammalian PI(4,5)P_2_-PLC (so-called PI-PLC) also hydrolyzes both PI(4,5)P_2_ and PI, its PI(4,5)P_2_-PLC activity is 30∼50-fold stronger than the PI-PLC activity (35, 54). Therefore, the substrate specificity of SMSr as PI-PLC for phosphoinositide is different from PI(4,5)P_2_-PLC. The PI(4,5)P_2_ levels in the mammalian cells are ∼100-fold lower than the PI levels (55). Moreover, the majority of PI in mammalian cells is distributed in the ER membrane (56), whereas PI(4,5)P_2_ predominantly localizes at the plasma membrane (57, 58). Taken together, even though SMSr as PI-PLC hydrolyzed PI(4,5)P_2_ *in vitro*, it is possible that SMSr proteins hydrolyze PI more strongly than PI(4,5)P_2_ because little or no 18:0/20:4-PIP_2_ exists in the ER membrane.

It has been suggested that PC-specific phospholipase C (PC-PLC) plays pivotal roles in signal transduction pathways that are involved in atherogenesis, inflammation, and carcinogenesis (12, 40, 59). However, the molecular entity of mammalian PC-PLC has not yet been identified since the activity was reported 40 years ago (44). Moreover, we reported that coimmunoprecipitates using an anti-DGKδ antibody from C2C12 myoblast cells contained PC-PLC activity and that DGKδ interacted with SMSr (30, 31). Furthermore, D609, a PC-PLC inhibitor (40) (Table 2), attenuated the PC-PLC activity of SMSr (Fig. 5*E*). Therefore, it is likely that SMSr is PC-PLC, which has been awaited for molecular entity identification for quite some time (∼40 years).

PE-PLC activity in mammalian cells was discovered approximately 30 years ago (16, 45, 46, 60) and is inhibited by D609 (13). Although bacterial and plant PE-PLC has been cloned, mammalian PE-PLC molecules have not yet been identified. In the present study, we, for the first time, found the mammalian protein that displays PE-PLC activity. Therefore, it is possible that SMSr is the previously reported PE-PLC (46).

We previously reported that in C2C12 myoblasts, DGKδ apparently prefers to phosphorylate palmitic acid (16:0) and/or palmitoleic acid (16:1)-containing DG molecular species, but not 20:4-containing DG delivered from the PI(4,5)P_2_ cycle under high-glucose stimulation (31). However, DGKδ enigmatically fails to possess such DG species selectivity *in vitro*. Intriguingly, we recently found that SMSr interacted with DGKδ *via* their SAMDs and provided 16:0 and/or 16:1-containing DG species to the enzyme (30). However, it remains unclear whether SMSr itself determines DG species that are supplied to DGKδ. In the present study, SMSr preferentially produced 16:0 and/or 16:1-containing DG species *in vitro*. Therefore, SMSr likely limits DG species utilized by DGKδ. Thus, the molecular machinery of the novel DG supply pathway independent of the PI(4,5)P_2_ cycle becomes clearer.

In summary, the current study, for the first time, showed that human SMSr displayed PAP, PI-PLC, PE-PLC, PG-PLC, and PC-PLC activities *in vitro*, in addition to CPES activity. In particular, PAP (∼300-fold), PI-PLC (∼10-fold), PC-PLC (∼3-fold), and PE-PLC activity (∼4-fold) were stronger than the CPES activity. Thus, beyond the enzyme’s previously established CPES activity, SMSr can be categorized as a new type of PLC with a broad head group specificity, *i.e.*, a multi-glycerophospholipid PLC hydrolase (MG-PLC). The current results allow us to propose the new DG/PA metabolic pathway “Luminal PA, PI, PC and PE → SMSr (MG-PLC) → DG → flip-flop → DGKδ → cytoplasmic PA” in the ER membrane, which is independent of the PI(4,5)P_2_ cycle (Fig. 8). Future studies exploring the enzymatic property of SMSr may contribute to understanding of the novel DG/PA metabolic pathway in the ER membrane and the pathogenetic mechanisms of various diseases related to DGKδ, type 2 diabetes (25, 26) and obsessive–compulsive disorder (27, 28) and, probably, to PC-PLC, atherogenesis, inflammation, and carcinogenesis (12, 40, 59).

## Experimental procedures

### Materials

Lipids: 1,2-dipalmitoyl-sn-glycero-3-phosphate (16:0/16:0-PA), 1-palmitoyl-2-oleoyl-*sn*-glycero-3-phosphoinositol (16:0/18:1-PI), 1-palmitoyl-2-oleoyl-*sn*-glycero-3-phospho-L-serine (16:0/18:1-PS), 1,2-dioleoyl-sn-glycero-3-phosphate (18:1/18:1-PA), 1-stearoyl-2-arachidonoyl-sn-glycero-3-phosphoinositol (18:0/20:4-PI), 1-stearoyl-2-arachidonoyl-sn-glycero-3-phospho-(1'-myo-inositol-4',5'-bisphosphate) (18:0/20:4-PI(4,5)P_2_), 1-stearoyl-2-arachidonoyl-sn-glycero-3-phosphate (18:0/20:4-PA), and 1-stearoyl-2-docosahexaenoyl-sn-glycero-3-phosphate (18:0/22:6-PA) were obtained from Avanti Polar Lipids. 1,2-dimyristoyl-sn-glycerol (14:0/14:0-DG), 1-palmitoyl-2-oleoyl-*sn*-3-phosphoglycerol (16:0/18:1-PG), and N-stearoyl-D-erythro-sphingosine (d18:1/18:0-Cer) were obtained from Cayman Chemical Company. 16:0/18:1-*sn*-glycero-3-phosphocholine (16:0/18:1-PC) was purchased from Sigma-Aldrich.

Detergents: n-dodecyl-β-D-maltoside (DDM) was obtained from Cayman Chemical Company. Cholesteryl hemisuccinate (CHS) was purchased from Sigma-Aldrich.

### Plasmids

Human SMSr and SMSr-ΔSAMD (amino acid residues 79–413) tagged with N-terminal 6×His or C-terminal V5 were previously made (30). 6× His-tagged SMSr and SMSr-ΔSAMD were subcloned into the pOET3 vector (Oxford Expression Technologies) at XbaI/XhoI sites.

### Antibodies

Rabbit polyclonal anti-His-tag antibody (PM032) was obtained from Medical and Biological Laboratories. Mouse monoclonal anti-V5 antibody (clone E10/V4RR, cat. no. MA5-15253) was obtained from Thermo Fisher Scientific. A peroxidase-conjugated goat anti-mouse IgG antibody was purchased from Bethyl Laboratories. A peroxidase-conjugated goat anti-rabbit IgG antibody (111-036-045) was obtained from Jackson Immuno Research.

### Cell culture

COS-7 cells were maintained on 150-mm dishes (Thermo Fisher Scientific) in Dulbecco’s Modified Eagle’s Medium (DMEM) (Wako Pure Chemicals) containing 10% fetal bovine serum (Thermo Fisher Scientific), 100 units/ml penicillin G (Wako Pure Chemicals), and 100 μg/ml streptomycin (Wako Pure Chemicals) at 37 °C in an atmosphere containing 5% CO_2_ as described (30). For quantitation of PA and DG levels, 1 × 10^6^ cells were plated on 100-mm dishes. After 24 h, plasmid cDNAs were transfected using PolyFect (Qiagen) according to the manufacturer’s instruction manual. After transfection, the cells were cultured for an additional 24 h and were used for the experiments.

Sf9 cells were maintained in Sf-900 II serum-free medium (Invitrogen) in sterile Erlenmeyer flask at 120 rpm and 28 °C without CO_2_ in the dark. Volume of the medium was kept at 20 to 30% of flask volume.

### Expression of SMSr *via* baculovirus-insect cell system

To generate recombinant baculovirus, the flashBAC system (Oxford Expression Technologies) and the pOET3 vector were used as described in flashBAC System Transfection Protocol (https://www.mirusbio.com/assets/protocols/flashbac-baculovirus-expression-systems-protocol.pdf) (61, 62). For generation of baculovirus (P_0_), 1.0 × 10^6^ cells were plated on 35-mm dishes. The DNAs (flashBAC and pOET3 vector) were transfected using TransIT-Insect Reagent (Mirus Bio) according to the manufacturer’s instruction manual. The baculoviral stocks (P_0_, ∼1 × 10^6^ pfu/ml) were harvested at 5 days posttransfection supernatants and passed through a 0.22 μm filter.

Baculoviral stocks were amplified for seven rounds of amplification at 2 × 10^6^ cells/ml, multiplicity of infection (MOI) of 0.1. Viral titer (plaque forming unit (pfu)/ml) was determined *via* plaque assay as described in flashBAC System Transfection Protocol. Viral stocks were stored at −80 °C until use.

To express recombinant proteins, Sf9 cells were seeded at 3 × 10^6^ cells/ml and infected cells with recombinant baculovirus at MOI of 2. Infected cells were harvested after 24 h and the pellets were resuspended in 40% (v/v) glycerol diluted in phosphate-buffered saline. The cell samples were flash-frozen in liquid nitrogen and stored at −80 °C until use.

### Preparation of Sf9 membranes containing overexpressed recombinant protein

The membranes of Sf9 cells containing overexpressed proteins were prepared *via* the protocol described by JCIMPT, an NIH Structural Biology RoadMap Initiative Project (https://commonfund.nih.gov/sites/default/files/JCIMPT_MembranePrep.pdf) (63, 64, 65).

Frozen Sf9 cells expressing recombinant proteins were thawed at 4 °C. After centrifugation at 10,000*g* for 30 min at 4 °C, cell pellets were resuspended in ice-cold hypotonic buffer (10 mM HEPES-NaOH (pH 7.5), 10 mM MgCl_2_, 20 mM KCl, 1 mM phenylmethylsulfonyl fluoride (PMSF), 20 μg/ml aprotinin, 20 μg/ml leupeptin, 20 μg/ml pepstatin and 20 μg/ml soybean trypsin inhibitor), and cells were homogenized on ice *via* homogenizer. After ultracentrifugation at 100,000*g* for 30 min at 4 °C, pellets were resuspended in ice-cold high-osmolarity buffer (10 mM HEPES-NaOH (pH 7.5), 1 M NaCl, 10 mM MgCl_2_, 20 mM KCl, 1 mM PMSF, 20 μg/ml aprotinin, 20 μg/ml leupeptin, 20 μg/ml pepstatin, and 20 μg/ml soybean trypsin inhibitor) and homogenized on ice *via* homogenizer. This procedure using high-osmolarity buffer was repeated twice. After ultracentrifugation at 100,000*g* for 30 min at 4 °C, Sf9 membranes were resuspended in 40% (v/v) glycerol diluted in high-osmolarity buffer and homogenized. The Sf9 membranes were flash-frozen in liquid nitrogen and stored at −80 °C until use.

### Purification of 6× His-tagged proteins

The 6× His-tagged proteins were partially purified *via* affinity chromatography. Frozen Sf9 membranes containing recombinant proteins were thawed at 4 °C. After ultracentrifugation at 100,000*g* for 30 min at 4 °C, the purified membranes were lysed *via* homogenization on ice with ice-cold lysis buffer (50 mM Tris-HCl, pH 8.0, containing 500 mM NaCl, 10% (v/v) glycerol, 1% (v/v) NP-40, 20 mM imidazole, 1 mM PMSF, 0.1 mM DTT, 20 μg/ml aprotinin, 20 μg/ml leupeptin, 20 μg/ml pepstatin, and 20 μg/ml soybean trypsin inhibitor) and incubated at 4 °C for 1 h with gently stirring *via* magnetic stirrer. The supernatant (1% NP-40 soluble fraction) was isolated by ultracentrifugation at 200,000*g* for 30 min at 4 °C, and then the supernatant was incubated with nickel–nitrilotriacetic acid (Ni-NTA) agarose (Wako Pure Chemicals) at 4 °C for 2 h with gently rotation. After centrifugation at 200*g* for 10 min at 4 °C, the Ni-NTA agarose was resuspended in lysis buffer and transferred into an empty column. The beads were washed with 30 ml of lysis buffer, followed by washing with 10 ml of wash buffer (50 mM Tris-HCl, pH 8.0, containing 500 mM NaCl, 10% (v/v) glycerol, 0.5% (v/v) NP-40, 50 mM imidazole, 0.1 mM DTT). Subsequently, the bound proteins were eluted with an elution buffer (50 mM Tris-HCl, pH 8.0, containing 500 mM NaCl, 10% (v/v) glycerol, 0.1% (v/v) NP-40, 500 mM imidazole, 0.1 mM DTT). The partially purified proteins were further purified *via* size-exclusion chromatography. After concentration using Amicon Ultra-15 (Merk), the partially purified samples were subjected to size-exclusion chromatography on an ENrich SEC 650 column (Bio-Rad equipped with a Bio-Rad NGC chromatography system). The column was equilibrated with 20 mM Tris-HCl (pH 7.4), 200 mM NaCl, 5% glycerol, 0.5 mM dithiothreitol, and 0.018% NP-40.

### Immunoblot analysis

The purified proteins were mixed with a 5×SDS sample buffer and incubated at 37 °C for 2 h instead of boiling. The samples were analyzed by SDS-PAGE and an immunoblot analysis, as described (30).

### Preparation of DDM/CHS stock solutions

To prepare detergents stock solution (10% (w/v) n-dodecyl-β-D-maltoside (DDM) and 2% (w/v) cholesteryl hemisuccinate (CHS)), 5 g of DDM was added into 40 ml of 200 mM Tris-HCl (pH 8.0), followed by gently rotation until DDM goes into solution. The detergent (1 g of CHS) was added to DDM solution and sonicated until solution becomes translucent. After sonication, 10 ml of 200 mM Tris-HCl (pH 8.0) was added and incubated at 25 °C with gently rotation until the solution becomes transparent. The detergents solution was stored at −25 °C until use.

### Preparation of micelles

The reaction solution for PLC and CPES activity assay was prepared as we reported (34). Phospholipids dissolved in chloroform/methanol (2:1 (v/v)) and d18:1/18:0-ceramide in chloroform were dried under N_2_ gas to yield a lipid film on the wall of the vial. The lipid film was resuspended in the 2×reaction buffer (40 mM Tris-HCl (pH 7.4), 30 mM KCl, 200 mM NaCl, 600 mM sucrose, 0.2% (w/v) DDM, 0.04% (w/v) CHS) to a final total lipid concentration of 0.1 mM each. The 2×reaction solutions containing lipids were vortexed for 3 min and sonicated in a bath sonicator (Sonifilter model 450) four times for 2 min at 65 °C.

### Mixed micellar assay

The DG-generating activities (PLC, PAP, or CPES) of purified proteins were evaluated *via* a DDM mixed micellar phospholipase C assay previously published (66, 67) with a modification. Because DDM/CHS (5:1) micelles provide a more membrane-like environment, we adopted DDM/CHS (5:1) for a mixed micellar DG-generating enzyme activity assay.

SMSr-containing samples (10 μl) were diluted in 10 μl ice-cold 2×reaction buffer on ice (final concentration of 20 mM Tris-HCl (pH 7.4), 15 mM KCl, 200 mM NaCl, 300 mM sucrose, 0.1% (w/v) DDM, 0.02% CHS, and 50 μM phospholipids (16:0/18:1-PA, 16:0/18:1-PC, 16:0/18:1-PE, 16:0/18:1-PI, 16:0/18:1-PS or 16:0/18:1-PG)). For a CPE synthase activity assay, d18:1/18:0-ceramide was added into the reaction buffer (final concentration of 50 μM). Because SMSr is membrane-bound and lipid-dependent enzyme, the surface dilution kinetic scheme was used (68, 69). The surface concentration of phospholipids in the micelles (1.87 mM DDM and 0.41 mM CHS mixed micelle) is 2.14 mol% (68). The mol % of lipids in DDM/CHS/lipid-mixed micelle was calculated by the formula: mol% = 100 × [lipid (mol)]/([DDM (mol)] + [CHS (mol)] + [lipid (mol)]).

After incubation (the enzyme reaction) for 2 h at 37 °C, lipids were extracted by Bligh & Dyer method (70), followed by quantitation of 16:0/18:1-DG using LC-MS/MS (30, 34).

### Statistical analysis

The data are represented as the means ± SD and were analyzed using Student’s *t*-test or one-way ANOVA using GraphPad Prism 8 (GraphPad).

## Data availability

All data are contained within the article.

## Conflict of interest

The authors declare no conflicts of interest associated with the contents of this article.
